# Purinergic Signaling in Ovarian Carcinoma

**DOI:** 10.34172/apb.025.46032

**Published:** 2025-10-25

**Authors:** Angélica Sofía Martínez-Ramírez, José David Nuñez-Ríos, Ana Patricia Juárez-Mercado, Anaí del Rocío Campos-Contreras, Francisco G. Vázquez-Cuevas

**Affiliations:** ^1^Laboratorio de Cultivo de Células Animales, Universidad de Papaloapan, Campus Tuxtepec, Circuito Central Colonia Parque Industrial, Tuxtepec Oaxaca, México; ^2^Laboratorio de Fisiología Celular, Instituto de Neurobiología-UNAM Campus Juriquilla. Boulevard Juriquilla, Juriquilla, Querétaro, México; ^3^Academia de Ingeniería en Biotecnología, Universidad Tecnológica de Corregidora, Corregidora, Querétaro, México; ^4^Facultad de Ingeniería, Universidad Autónoma de Querétaro, Querétaro, México

**Keywords:** Ovarian carcinoma, Purinergic receptors, Extracellular ATP, Adenosine

## Abstract

Ovarian carcinoma (OC) is the most lethal gynecological cancer worldwide. Around 95% of patients exhibit recurrence five years after treatment; 80% experience recurrence within 18 months after first-line treatment, and progression-free survival rates have not changed over the past 40 years. New therapeutic approaches are imperative to face this complex disease. The purinergic system is a newly recognized element of the tumor microenvironment (TME), as it exhibits a pro-tumor role. Tumor cells release adenosine triphosphate (ATP) into the TME, where it exerts autocrine-paracrine actions that regulate several processes, including the induction of a metastatic phenotype, cell proliferation, and metabolic adaptations. In the extracellular milieu, ATP is converted to adenosine (ADO) by ectonucleotidases (CD39 and CD73), thereby significantly blocking the anti-tumor immune response through interactions with various immune cells. Recent analyses have focused on the diversity and plasticity of purinergic signaling in OC. This review outlines the disease, explains basic concepts of purinergic signaling, and summarizes experimental evidence that indicates purinergic elements may serve as potential targets for novel therapies to overcome OC.

## Introduction

 Ovarian carcinoma is the most lethal gynecological malignancy worldwide, with a mortality rate of 64%, a percentage notably higher than that observed in breast cancer (29%).^1^ Approximately 80% of the patients experience disease recurrence within 18 months after initial treatment.^2^ In this context, it is imperative to identify new therapeutic targets to enhance the efficacy of conventional therapies.

 The purinergic system has emerged as a key intercellular communication system within the tumor microenvironment (TME) in several cancer types. Accumulating evidence demonstrates that purinergic substances such as ATP and adenosine (ADO) play two major roles within the TME: 1) they exert autocrine actions on tumor cells, regulating fundamental processes such as proliferation, migration and epithelial-to-mesenchymal transition (EMT); and 2) they mediate interactions between tumor and immune cells, thereby suppressing anti-tumor immune response. In this work, we systematically reviewed the available information on purinergic signaling elements as potential targets in ovarian carcinoma. Throughout the text, we present an overview of OC, described the purinergic system and its established role in cancer biology, reviewed the state of art of purinergic signaling in OC, and conducted database analysis to highlight the translational relevance of purinergic receptors.

## Ovarian carcinoma

###  Genetic and epidemiological data

 The Global Cancer Observatory (GLOBOCAN)^1^ recently reported that ovarian cancer (OvCa) is among the first five most frequent gynecological cancers, with 324,603 new cases and 206,956 deaths recorded in 2022. Although breast and cervical cancers have the highest incidence, OvCa is the most lethal, with a 64% mortality rate, in contrast to 29% for breast cancer and 53% for cervical cancer.^1^ The adjusted diagnosis rate is lower for underdeveloped countries than developed countries (5.0 and 9.1 per 100,000 people, respectively). However, it is still uncertain whether this difference is real or a result of underdiagnosis in developing countries. Despite a recent global decline in OC incidence, lethality rates have not changed since the 1980s. GLOBOCAN projections suggest this trend will continue until 2045.^1^ The five-year survival rate for OvCa patients is below 50%, with 80% of these patients experiencing relapses 18 months after concluding primary treatment.^2^

 Studies have identified several risk factors for OvCa, including genetic and environmental factors, age, hormone treatments, number of pregnancies, and infertility.^3^ Moreover, diverse mutations in specific genes have been associated with OvCa risk, with the most extensively studied and well-characterized being the *BRCA1* and *BRCA2 loci; *these arepresent in 17% of patients. Other genes encode for proteins related to DNA repair, such as *RAD51C, BRIP1, BARD1, *and* PALB2.*^4^

###  Classification of ovarian carcinoma 

 Since the 1930s, pathologists have described OvCa as a group of distinct diseases. OvCa is typically classified according to the tissue of origin into epithelial, germinal and stromal subtypes, which are highly heterogeneous in both prevalence and clinical prognosis. The most common type is epithelial ovarian carcinoma (OC), accounting for 90% of all diagnosed cases.^5^ This review will focus on OC due to its prevalence.

 Histopathologically, OC is further sub-classified as serous, mucinous, endometrioid, and clear-cell. Serous tumors are characterized by frequent nuclear atypia, mitotic bodies, crypt-shaped spaces, and large nuclei; mucinous tumors have mucin-containing columnar cells; endometrioid tumors exhibit a glandular phenotype with pseudostratified columnar cells, nuclear atypia, and variable grades of stroma; and clear-cell tumors are characterized by cleared cytoplasm, giant cells, nuclear atypia, and interspersed hyaline zones.^5^

 Robust molecular methods have revealed differences in the expression patterns of molecular markers in specific tumors; for instance, low-grade serous tumors exhibit BRAF and KRAS mutations, whereas high-grade serous tumors do not. These findings led to the creation of a new classification for ovarian epithelial tumors based on their gene expression patterns, differentiation grade, and response to therapy. Type I tumors comprise low-grade serous, low-grade endometrioid, clear-cell, mucinous, and transitional cell carcinomas. They exhibit KRAS, BRAF, and ERBB2 mutations and less frequent TP53 mutations. These tumors are characterized by low invasiveness and are associated with a favorable prognosis. Type II tumors include high-grade serous, undifferentiated, and mixed malignant mesodermal carcinomas.^6^ These tumors harbor TP53 mutations ( > 95% of the cases), loss-of-function mutations in BRCA1/2, and less frequent KRAS, BRAF, and ERBB2 mutations. Type II tumors are extremely invasive, have unfavorable prognosis, show a limited response to treatment, and have high recidivism. High-grade serous OC (HGSC) constitutes approximately 75% of tumor type II cases.^6^ The histological and molecular diversity among OC tumors demonstrates the complex nature of this pathology, highlighting the need to find new markers, clinical predictors, and therapeutic targets.

###  Origin of ovarian carcinoma 

**Figure 1:**
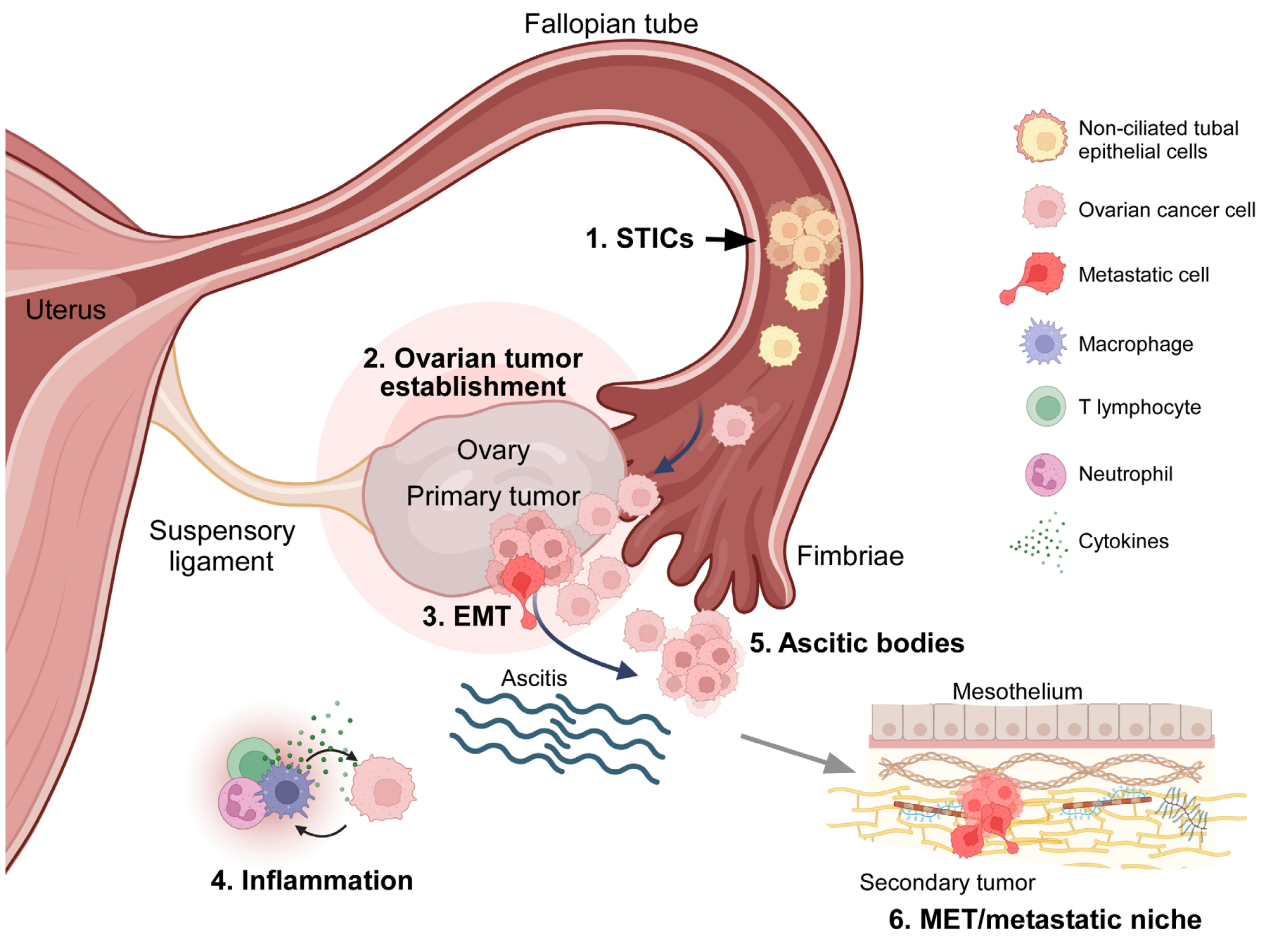
The origin of high-grade serous ovarian carcinoma (HGSC) and transcoelomic dissemination mechanism. 1.- Tumor cells originate in the Fallopian tube epithelium and form serous tubal intraepithelial carcinomas (STICs); tumor cells detach from and colonize the ovary. 2.- HGSC colonizes the ovarian tissue and forms the primary tumor. 3.- Tumor cells undergo epithelial-to-mesenchymal transition (EMT), detach the main tumor, and spread to the peritoneal cavity. 4.- Inflammation produced by cancer cells in the peritoneal cavity induces liquid accumulation (ascitis). 5.- Within the peritoneum, metastatic cells avoid anoikis and generate ascitic bodies. 6.- Tumor cells reach the mesothelium and adjacent organs and undergo mesenchymal-to-epithelial transition (MET), developing the metastatic niche. Created in BioRender. https://BioRender.com/1cgfv55

 Tracking the cellular origin of OC poses a significant challenge, mainly due to late-stage diagnosis. HGSC, for example, is commonly diagnosed at an advanced stage, when it has spread throughout the abdominal cavity. Early studies proposed that all OCs originate from the ovarian superficial epithelium (OSE).^7^ Additionally, evidence suggests that type II OC tumors originate from the fallopian tube epithelium. Observations in patients who underwent prophylactic salpingo-oophorectomy and exhibited loss-of-function mutations in BRCA1/2 genes support the latter hypothesis. The histopathological analysis of tissue collected from these patients revealed proliferative lesions identified as serous tubal intraepithelial carcinomas (STICs).^6^ STICs consist of non-ciliated tubal epithelial cells with cancerous features, such as loss of cell polarity, significant nuclear atypia, loss-of-function mutations in TP53, and a high mitotic index, as indicated by an elevated Ki67 signal. Some authors propose that STICs precede HGSC, as they are detached from the fallopian tube epithelium and colonize the ovary (Figure 1). In genetic expression studies, researchers have found that HGSC cells are more similar to fallopian tube epithelium cells than to OSE cells,^8^ suggesting that STICs are precursors to HGSC.

###  Transcoelomic dissemination of ovarian cancer 

 Unlike other malignant tumors, OC does not spread through blood or lymphatic vessels; instead, it utilizes a mechanism known as transcoelomic dissemination. This term denotes the invasion of organs in the pelvic-abdominal cavity within the peritoneum after the exfoliation of cancer cells.^9^ During this process, primary tumor cells display phenotypic changes, including EMT, thereby acquiring mesenchymal characteristics that induce detachment to the peritoneal cavity. Most of these cells die due to the lack of contact with the extracellular matrix (anoikis), but surviving cells form clusters called ascitic bodies, which are typical features of OC. Ascitic bodies are heterogeneous clusters containing cancer cells, dendritic and epithelial cells, T and B lymphocytes, fibroblasts, and macrophages.^9,10^ These cell clusters are driven by the movement of fluid inside the peritoneal cavity. Secondary tumors may develop when ascitic bodies spread to other organs, including the fallopian tubes, uterus, liver, rectum, bladder, pelvic wall, and omentum (Figure 1). Researchers have proposed that the accumulation of ascitic bodies is induced by increased vascular and lymphatic permeability, which can be prompted by tumor cells inside the peritoneal cavity. Therefore, the identification of ascitic bodies during cytoreductive surgery after neoadjuvant chemotherapy is indicative of a poor prognosis.^10^

 The establishment of a metastatic niche requires molecular interaction between metastatic cells and the mesothelium of the target organ. *In vivo* and *in situ* studies have found that this interaction occurs, at least partially, between the glycoprotein CD44—expressed in tumor cells within ascitic bodies—and hyaluronic acid in the mesothelial extracellular matrix.^11^

## Purinergic system and its role in cancer

###  Nucleotides as cellular messengers

 Nucleotides are versatile molecules. They serve as energy transfer molecules and building blocks for nucleic acids, while also functioning as crucial intercellular messengers. In 1929, Drury and Szent-Györgyi obtained experimental data showing adenine molecules as signaling molecules.^12^ Furthermore, through the use of nucleotide-rich extracts, the authors observed physiological events in the cardiovascular system, such as a decreased heart rate and blood vessel dilation. In 1959, Pamela Holton demonstrated the release of adenosine triphosphate (ATP) by electric stimulation of the sensory nerve in a rabbit ear. However, the term “purinergic” was not used until 1972, when it was proposed by Geoffrey Burnstock. In his studies on the nerve responses at the neuromuscular juncture between sympathetic nerves and the vas deferens muscle of guinea pigs, Burnstock described a non-cholinergic and non-adrenergic neurotransmitter system with ATP as its messenger.^13^ His hypothesis was rejected for decades but began to gain acceptance when purinergic receptors were cloned in the early nineties.^14^ Over the years, research has shown that ATP not only serves as a neurotransmitter but also as an autocrine or paracrine messenger released through diverse mechanisms.

**Figure 2:**
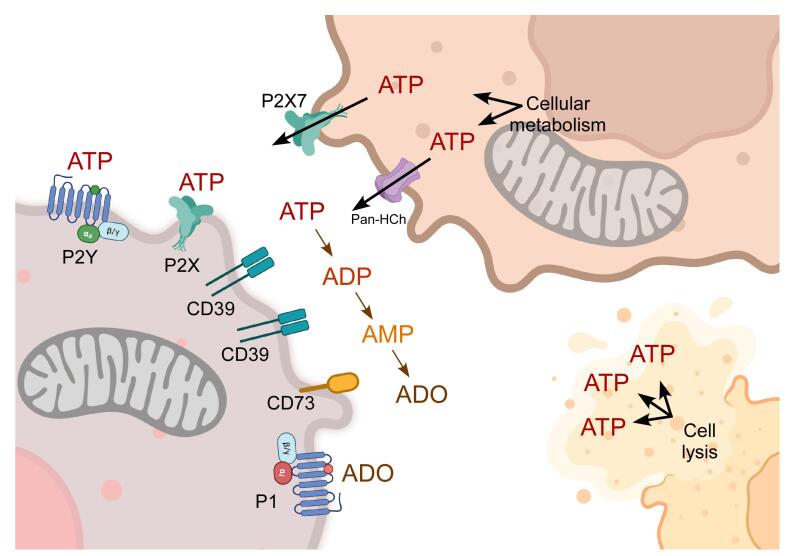
The purinergic system of intercellular communication. ATP is generated through metabolic pathways including glycolysis, oxidative phosphorylation, and beta-oxidation. ATP can be released into the extracellular milieu through different mechanisms, such as pannexin hemichannels (Pan-HCh), the P2X7 channel, and cellular lysis. Once in the extracellular milieu, ATP is hydrolyzed by ectonucleotidases (CD39 and CD73), generating the derivatives ADP, AMP, and ADO. AMP and ADO activate their receptors P2X, P2Y, or P1, thereby regulating specific transduction pathways. Created in BioRender. https://BioRender.com/gl1rdix

 The messenger molecules of the purinergic system are ATP, its dephosphorylated derivative ADP, and the nucleoside ADO. Furthermore, evidence demonstrates that uridine triphosphate (UTP) and diphosphate (UDP) can be released into the extracellular milieu and function as natural agonists for purinergic receptors. The elements completing this system are membrane-specific receptors for these messengers and ectonucleotidases that regulate the extracellular concentration of these molecules (Figure 2).^15^ ATP is released into the extracellular milieu through pannexin and connexin hemichannels, maxi-anion channels, volume-regulated ion channels, ABC transporters, purinergic receptor P2X7, vesicle secretion, and cellular lysis.^16^ Once in the extracellular milieu, ATP activates purinergic P2 receptors through autocrine and paracrine mechanisms. Additionally, membrane ectonucleotidases hydrolyze ATP, resulting in the formation of ADP, AMP, and ADO. Ectonucleotidases are divided into four families: CD39/E-NTPDases (ectonucleoside triphosphate diphosphohydrolases), E-NPPs (ectonucleotide pyrophosphatases), APs (alkaline phosphatases), CD73/ NT5E (ecto-5’-nucleotidase). Each family has distinct characteristics, which are outlined in Figure 2.^15,17^

 Purinergic receptors are categorized into two families: P1 receptors, which are sensitive to ADO; and P2 receptors, which are sensitive to adenine and uridine nucleotides. P1 receptors are G protein-coupled receptors (GPCRs), and A1, A2A, A2B, and A3 are their subtypes. A1 and A3 are preferentially coupled to Gi/0 proteins, whereas A2A and A2B are preferentially coupled to Gs. On the other hand, P2 receptors can be either GPCRs (P2Y) or ligand-activated ion channels (P2X).^15^ P2X receptors allow Na^+^ and Ca^2+^ inflow and K^+^ outflow. Seven genes encode different P2X subunits (P2X1-7), which conjugate forming homotrimeric or heterotrimeric complexes to constitute functional P2X receptors. The endogenous ligand for P2X receptors is ATP. On the other hand, P2Y receptors are also classified based on their functional characteristics. P2Y1, P2Y2, P2Y4, P2Y6 and P2Y11 are similar to P2Y1, whereas P2Y12, P2Y13 and P2Y14 are analogous to P2Y12. The fundamental difference between the two groups is their preferential coupling to heterotrimeric G proteins. The first group is preferentially coupled to Gq, and the second group is preferentially coupled to Gi. These receptors can be activated by ATP, ADP, UTP, UDP, and UDP-glucose.^15^

###  Release and processing of ATP within the tumor microenvironment 

 Tumors consist not only of cancer cells but also of an extracellular matrix, blood vessels, immune cells, and fibroblasts. Collectively, these elements form the tumor interstitium, which provides structural support and enables the diffusion of nutrients and oxygen within the tumor. The TME includes these interstitial components and the extensive array of chemical messengers they release.^18^

 Purinergic signaling is a crucial component of the TME, as it contains high concentrations of ATP, typically in the hundreds of micromolar range.^19^ Cell death leads to the enrichment of ATP, with its release being regulated by tumor, immune, and stromal cells in the interstitium. ATP release into the extracellular milieu has been demonstrated in cell lines from different cancer types, including lung, bladder, breast, and OC. The Francesco Di Virgilio group in Ferrara, Italy, provided definitive *in situ* evidence that the TME enriches ATP concentration. To monitor ATP tumor concentration in the whole animal, the Di Virgilio group developed a luciferase variant. This luciferase variant, which is a protein that emits light in response to ATP binding, was fused with a transmembrane segment of another protein. The luciferase catalytic region in this chimeric protein was placed on the extracellular side of the membrane, enabling its activation in the presence of extracellular ATP. The chimera was expressed in reporter cells that were injected into the tail vein in mice with an OC cell xenotransplant (OVCAR-3). Once the reporter cells were distributed throughout the organism, the luminescence level in the whole animal was measured. Results indicated that the TME contained high ATP concentrations (hundreds of millimolar), while healthy tissue showed ATP concentrations that were 10000 times lower.^19^

 Extracellular ATP in the TME functions as a substrate for ectonucleotidases, resulting in a direct source of extracellular ADO which also enriches this nucleoside. Micro-dialyzed samples reveal higher concentrations of ADO in the center of the tumor than in external areas or healthy tissues.^20^ Since the activity of hypoxia inducible factor (HIF-1α) promotes the expression of ectonucleotidases CD39 and CD73, research has suggested that oxygen deficits in tumors favor ADO production.^21^ Furthermore, CD39 and CD73 expression has been demonstrated in tumor cells, immune cells, and stroma cells, indicating that most TME cells contribute to the modulation of purinergic ligand levels and composition within the tumor.^22^

###  Purinergic messenger activity in cancer

 There is ample evidence that nucleotides, mainly ATP and ADO acting through P1 and P2 receptors, modulate acquired features of cancer cells that facilitate uncontrolled proliferation, neighboring tissue invasion, and dissemination to distant organs.^22^ In our opinion, these actions can be classified into two levels: 1) as mediators of the functional interaction between cancer cells and immune cells within the TME and 2) as autocrine-paracrine messengers that regulate diverse processes within tumor cells.

###  Purinergic messengers and cellular interactions in the TME

 The purinergic system serves as an immune modulator, influencing the interaction interphase between cancer and immune cells. Extracellular ATP functions as a chemoattractant for both innate and adaptive immune cells, while ADO acts as an immunosuppressant, changing immune cell phenotypes and dismantling the antitumor immune response.^21^

 In the context of tissue damage, ATP released by dying cells functions as a damage-associated molecular pattern (DAMP). DAMPs are metabolites whose localization and/or compartmentalization changes due to alterations in tissue conditions. This change serves as an alarm signal to recruit macrophages, neutrophils, and T cells. During antitumor therapies, dying cells release large amounts of ATP into the extracellular milieu, which recruits circulating immune cells and concurrently activates signaling mechanisms in infiltrated immune cells. For instance, in dendritic cells, the activation of P2X7 receptor by extracellular ATP induces NLRP3 inflammasome assembly and, therefore, the release of interleukin-1β (IL-1β), a pro-inflammatory cytokine that acts on lymphocytes.^23^ Chemotherapy with oxaliplatin and anthracyclines is inefficient in P2X7 receptor knockout mice (P2X7^-/-^) or mice lacking components of the Nlrp3 inflammasome, such as caspase 1 (casp^-/-^). This suggests that the inflammatory response triggered by P2X7 is crucial for the success of this therapy.^24^

 As previously mentioned, the increment in extracellular ATP concentration induces a concomitant rise in ADO concentration. ADO has significant immunosuppressive effects; for example, ADO formation by OC cells attracts myeloid lineage cells and induces their differentiation into M2-TAMs (tumor-associated macrophages). M2-TAMs exhibit a non-inflammatory phenotype, are non-phagocytic, and play a protective role against the antitumor immune response.^25^ Moreover, M2-TAMs express the enzymes required for ADO production, thereby sustaining the prevalence of this phenotype in circulating macrophages infiltrating the TME.^25^ ADO production has also been observed in other TME resident immune cells, including regulatory T cells (Tregs) and cytotoxic T lymphocytes (CD8^+^T cells).^26^ Evidence suggests that the effects of ADO on the immune cell phenotype are mediated by A2A ADO receptors, as their genetic deletion or pharmacological inhibition facilitates tumor rejection driven by CD8^+^T cells.^27^ Together, ATP and ADO reconfigure interactions between tumor and immune cells to evade the antitumor immune response.

####  Autocrine-paracrine actions mediated by the purinergic system within the TME

 In addition to mediating the interactions between tumor and immune cells, ATP within the TME can function in an autocrine-paracrine manner through its specific receptors. One of the first purinergic receptors to attract attention in biomedical research was the P2X7 receptor because of its ability to induce cell death in various cell types. Researchers have hypothesized that this receptor acts as a tumor suppressor, as some cancer types, including endometrial cancer, exhibit a significant reduction in P2X7 expression levels in the cancerous tissue.^28^ Paradoxically, analysis of tumor tissues from other organs (e.g., breast or prostate) revealed that the P2X7 receptor was overexpressed and associated with high tumor grade and low survival prognosis.^29,30^ Additionally, cellular studies in these tissues showed that the P2X7 receptor promotes tumor growth by different signal transduction pathways, and it is a positive regulator of aerobic glycolysis.^31^ The P2X7 receptor is activated with high ATP concentrations (100 uM to 1 mM). However, the concentrations of ATP within the TME are sufficient to trigger its activation.^19^ The dual role of the P2X7 receptor as an apoptosis inducer and a proliferation promoter is attributed to alternative splicing variants that significantly alter the signaling mechanisms regulated by this receptor.^19,32,33^

 On the other hand, P2Y2 has been reported to play a role in the acquisition of metastatic characteristics of tumor cells. Several research groups have demonstrated that the P2Y2 receptor modulates cell migration and EMT in both healthy and transformed cell types.^34-36^

 In addition, purinergic signaling modulates metabolic adaptations in cancer cells, resulting in a more efficient production of ATP. This metabolic phenomenon, known as the Warburg effect, favors aerobic glycolysis and lactate formation over pyruvate production. For instance, in HEK293 cells activation of heterologous expressed P2X7 receptors promotes the Warburg effect. In the ACN neuroblastoma cell line, the P2X7 receptor upregulates the expression of glycolytic enzymes and other proteins, including glucose transporter 1, enzyme glyceraldehyde phosphate dehydrogenase, phosphofructokinase, pyruvate kinase 2, and pyruvate dehydrogenase, illustrating how the purinergic system contributes to the metabolic adaptations in cancer cells.^31^

## Purinergic signaling in ovarian carcinoma

###  P2Y2 receptor in ovarian carcinoma cells

 The first study analyzing the effects of P2Y receptor activity in OC cells was published in 2000.^37^ This research showed that stimulation of EFO-21 and EFO-27 cells with purinergic agonists elicits changes in intracellular Ca^2+^ concentration ([Ca^2+^]_i_). The pharmacological profile of these responses indicated that they depend on P2Y2 receptor activation, whose expression was confirmed by reverse transcription and polymerase chain reaction (PCR). Elicited responses exhibited two components: a fast peak that depended on the release from intracellular Ca^2+^ storages and a sustained component involving Ca^2+^ influx. Functional experiments performed in the same study showed that incubation of cultures with the non-hydrolyzable analog of ATP, ATPγS, decreases the proliferative effect induced by fetal bovine serum, suggesting an antiproliferative role for the P2Y2 receptor.

 On the other hand, P2Y2 receptor expression was also demonstrated in the IOSE29 (preneoplastic), IOSE-29EC (neoplastic), and OVCAR-3 (metastatic) cell lines. Stimulation of the receptor with ATP induced cell proliferation throughout a pathway involving protein kinase C and the mitogen-activated protein kinase ERK.^38^

 Research has also examined the participation of P2Y receptors in the regulation of cell migration and invasion in OC. In the SKOV-3 metastatic cell line, stimulation with 100 μM of UTP (a P2Y2 and P2Y4 receptor agonist) increased cell migration through a pathway that involved EMT induction and the transactivation of epidermal growth factor receptor. Although SKOV-3 cells express both P2Y2 and P2Y4 subtypes, the knockdown of P2Y2 abolished the effect, thereby confirming a role for this receptor. EMT induction was probed by the increased expression of the intermediate filament fiber Vimentin and the SNAIL transcription factor, as well as the downregulation of E-cadherin.^39^ These observations indicated that the P2Y2 receptor induces the mesenchymal phenotype in SKOV-3 cells, favoring metastatic characteristics. Additional studies identified other effectors of the P2Y2 receptor in SKOV-3 cells in the same cellular process, demonstrating that the K^+^ channel KCa3.1 activates downstream of P2Y2 in the regulation of cell migration.^40^

 Initial studies in EFO-21 and EFO-27 suggested that P2Y2 receptor stimulation induced the capacitive current of Ca^2+^ influx (a sustained component of the response).^37^ Recently, it was shown that P2Y2 receptor stimulation with 100 μM of UTP induces store-operated calcium entry (SOCE) in metastatic SKOV-3 cells but not in non-metastatic CAOV-3 cells, suggesting that SOCE activation is a hallmark of the invasive phenotype. Furthermore, the pharmacological inhibition of SOCE in SKOV-3 cells reduces the increase in cell migration triggered by P2Y2 receptor activation.^41^ These observations reinforce the previously described role of SOCE in sustaining the metastatic characteristics of breast cancer cells.^42^

###  P2X7 in ovarian carcinoma cells

**Figure 3:**
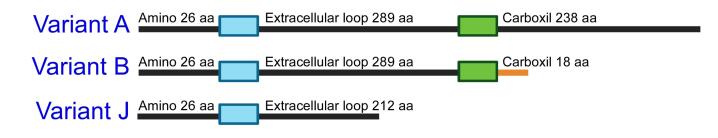
Structural differences between variants A, B, and J of the P2X7 receptor. Schematic representation of the distribution of the main domains in P2X7A, P2X7B, and P2X7J variants

 The P2X7 receptor was originally described as cytotoxic. However, ample evidence has revealed its pro-tumoral role in several tissue cancers. This paradox has been explained by the existence of alternative splicing variants, some of which lack the C-terminal end, thereby altering the receptor’s ability to induce cell death.^33^ The structural comparison of variants A, B, and J can provide clarity to this idea. Variant A (wild type) consists of two transmembrane segments, a short intracellular N-terminal end (26 amino acids [aa]), a large extracellular loop (289 aa), and a long intracellular C-terminal end (238 aa). Variant B has only 18 aa in the C-terminal end due to retention of an intronic sequence. For its part, variant J lacks the second transmembrane and conserves only a partial sequence of the extracellular loop (212 aa)^43^ (Figure 3). Functionally, variant A induces apoptosis by interacting with other proteins through the C-terminal end, while variant B loses this ability, and variant J acts as a dominant negative.^43^

 Another explanation is that the P2X7 receptor has differential activation levels, meaning that the cellular effects vary depending on whether the receptor is partially or fully activated.^32^

 The first observation of P2X7 expression in an OC biopsy was in a study investigating the carcinogenic and proliferative roles of this receptor in embryonic HEK293 cells. The study demonstrated the upregulated expression of P2X7 in a group of biopsies from different sources.^44^ A subsequent study analyzed nine tumor samples of diverse histopathological subtypes including endometrioid, clear cell, mucinous, high-grade serous, borderline, and control.^45^ In all the OC biopsies, the P2X7 receptor was upregulated and localized mainly in the tumor regions, whereas in the non-cancerous tissue, its expression level was low and limited to the ovarian surface epithelium. This finding suggested that P2X7 receptor upregulation promotes tumor progression. In the same study, the P2X7 receptor activity was assessed *in vitro* in the metastatic SKOV-3 cell line by RT-PCR, western blot, and immunofluorescence. Stimulation with the selective agonist BzATP induced a Ca^2+^ influx that was blocked by the antagonist A438079. An antibody microarray assay against the phosphorylated isoform of kinases in signal transduction revealed that P2X7 receptor stimulation leads to the phosphorylation of JNK, ERK and AKT, suggesting a role in cell proliferation and migration. Furthermore, cell viability decreased when the P2X7 receptor was blocked using the antagonist AZ10606120, when the negative dominant of P2X7 (E496A) was expressed, or when apyrase was added to the culture media for extracellular ATP hydrolysis.^45^

 AKT activation by BzATP in SKOV-3 cells suggested that P2X7 receptor stimulation may be associated with the regulation of cell migration. A recent study showed that P2X7 receptor activity supports the mesenchymal phenotype of SKOV-3 cells, since its pharmacological inhibition or genetic knockdown reduced cell migration and vimentin levels and increased transepithelial resistance and E-cadherin expression.^46^ In the same study, the P2X7 receptor was detected by western blot in a preparation of plasma membrane proteins obtained through biotinylation with a non-permeant reactive and precipitation with avidin-sepharose. An antibody targeting the extracellular loop of the receptor recognized a protein of ~45 kDa, while an antibody directed against the C-terminal end of the receptor revealed a faint band of ~75 kDa. The result suggested that two variants coexist in SKOV-3 cells: a wild-type variant (~75 kDa) and a ~45 kDa variant lacking the C-terminal end, which may be responsible for the effects of P2X7 on the phenotype of these cells.^46^

 On the other hand, it is well established that extracellular ATP, through the P2X7 receptor, can elicit the assembly of the NLRP3 inflammasome.^47^ In OC patients, NLRP3 inflammasome and P2X7 receptor are overexpressed in omental adipocytes. This finding suggests that the P2X7-NLRP3 complex produces a proinflammatory microenvironment at sites where secondary tumors will develop in advanced stages of the disease.^48^

 The data presented above indicates a pro-tumor role for P2Y2 and P2X7 receptors. Although no specific experiments have been designed to test a crosstalk between both receptors in OC, findings in other systems can shed some light about a possible interaction. A potential mechanism is a cooperative process of ATP release that results in the sequential activation of both receptors. It has been demonstrated that metastatic OC cells release ATP^49^ in sufficient quantities to stimulate P2Y2 receptors and trigger the release of more ATP through pannexin 1 hemichannels, thus increasing the extracellular concentration of the nucleotide.^50^ ATP concentration then becomes high enough to activate the P2X7 receptor and unleash its cellular effects. A similar mechanism has been described in macrophages.^51^ On the other hand, when cell death occurs in the tumor (e.g., during anticancer therapy), the extracellular concentration of ATP exhibits a significant increase^24^. Therefore, based on these findings, we reasoned that the extracellular level of ATP can activate P2Y2 and P2X7 in parallel in tumor cells, simultaneously triggering the downstream effects of both receptors.

###  Adenosine and CD73

 ADO exhibits autocrine-paracrine signaling in the TME and counteracts the mesenchymal phenotype of metastatic OC cells. SKOV-3 cells express ADO receptors A1, A3, and A2B, the ENPP1, CD73/NT5E, and liver alkaline phosphatase. Ectonucleotidase activity experiments revealed that SKOV-3 cells efficiently convert ATP into ADP and AMP into ADO but not ADP into AMP, limiting ADO production.^52^ These experiments also demonstrated that the extracellular ATP hydrolysis using apyrase facilitated ADO synthesis. This led to a decrease in both cell migration and the expression of EMT cell markers, thereby favoring an epithelial-like phenotype. Pharmacological manipulations that block ADO production, such as CD73 inhibition with 5’-(α,β-methylene)diphosphate (APCP), abolished these effects, which were mimetized by strategies that boost ADO accumulation in the extracellular space; for example, the inhibition of adenosine deaminase or ADO transporters.^52^ These findings suggest that the deficient conversion of ADP into AMP and, consequently, the blocking of ADO synthesis in SKOV-3 cells represent a phenotypic checkpoint, highlighting the relevance of ectonucleotidases in the acquisition of invasive characteristics.

 The role of the A2B receptor has been analyzed in SKOV-3 cells. A2B signaling counteracts the mesenchymal phenotype, resulting in decreased cell migration in the presence of stress fibers and increased E-cadherin expression.^53^ It has been demonstrated in breast cancer cells that cAMP, through PKA activation, induces mesenchymal-to-epithelial transition (MET).^54^ This mechanism is potentially also present on OC cells because the A2B receptor is coupled to cAMP production; however this hypothesis needs to be investigated. On the other hand, A2B receptor expression is enhanced in OVCAR-3 cells that are resistant to olaparib, a new chemotherapeutic drug. The activation of this receptor has been found to induce cell survival and migration through a pathway that involves IL-6 and Stat-3 activity.^55^ The antagonistic effects of the A2B receptor in SKOV-3 and olaparib-resistant OVCAR-3 cell lines demonstrate that the selection process for acquiring resistance to chemotherapeutics reveals populations of cells with differential phenotypes. In this case, the resistance to olaparib appears together with biased signal transduction pathways downstream of the A2B receptor. The changes in olaparib-resistant cells have not been fully elucidated and warrant further investigation. However, it is clear that adenosinergic receptors are altered in chemoresistant populations of cancer cells.

 CD73 and CD39 expression has been demonstrated in human OC biopsies. Immunohistochemical detection showed that both ectonucleotidases are highly expressed in cancer samples compared to non-cancerous ovarian tissues. Similar findings were observed for CD4^+^ and CD8^+^T-cell infiltration. Furthermore, primary cultures of ascitic fluid-derived cells (i.e., OaW42 and SKOV-3) also expressed CD39 and CD73. In co-cultures of OaW42 and SKOV-3 cell lines with human CD4^+^T-cells, abolishing or pharmacologically inhibiting CD39, CD73, or the A2B receptor resulted in T-cell proliferation blockage and inhibition of priming against cancerous cells. This effect was dependent on ADO production.^56^

 Data from the Australian Ovarian Cancer Study (GSE9899) revealed that elevated CD73 expression is associated with low patient survival rates, and high levels of CD73 gene expression are correlated with the EMT transcript signature.Complementary* in vitro* experiments have shown that extracellular ADO and CD73 support SKOV-3 cell proliferation. Furthermore, *in vivo* approaches show that co-injection of ID8 mouse OC cells and fibroblasts that overexpress CD73 in mice promotes immune evasion of tumor cells by inhibiting tumor-specific CD8^+^T-cell responses.^57^

 The combined effect of inhibiting CD73 and NAMPT (nicotinamide phosphoribosyltransferase), an important enzyme for NADH synthesis, has been assayed in an *in vivo* model of OC. This dual inhibition is supported by the fact that CD73 converts extracellular NAD^+^ to nicotinamide riboside, which enters the cell and boosts intracellular NAD^+^ synthesis. Dual treatment decreased ATP and NAD^+^ levels in the tumor and reduced tumor growth, as evidenced by Ki67 immunostaining. Furthermore, the mouse group receiving the therapy exhibited an increased survival rate.^58^ The study demonstrates that pharmacological inhibition of CD73 is a feasible strategy to potentiate the anti-tumor actions of NAMPT inhibitors.

 Growing evidence suggests that cancer stem cells are essential in tumorigenicity, having been identified as tumor initiating cells (TICs).^59^ Moreover, CD73 expression supports stemness maintenance in OC cellular populations. Therefore, pharmacological inhibition of the ectonucleotidase decreases cell spheroid formation, tumorigenicity, and the expression of stemness markers, and EMT-related transcript expression.^60^

 ADO in the TME constitutes an immunological checkpoint targeting immune system cells that results in the inhibition of the anti-tumor immune response.^21^ A meta-analysis of transcriptomic data from the MetaGxOvarian dataset found that high expression levels of CD39 and CD73 ectonucleotidases, together with an ADO-dependent gene signature in patients with HGSC, are associated with a poor prognosis for patient survival.^61^ Elevated expression of both enzymes was also related to chemoresistance. Furthermore, studies at protein level determined that cancer-associated fibroblasts express CD39 and CD73, contributing to the immune escape and chemoresistance of tumor cells.^62^ A recent study analyzed CD3^+^T-cells from peripheral blood, ascitic bodies, and tumor tissue in OC patients; the populations from ascitic bodies and tumor tissue were enriched in CD8^+^T-cells and exhibited a PD-1^high^, CD39^+^, and CD73^+^ phenotype and reduced expression of TCF-1, indicating decreased memory formation and effector capability, and high expression of TOX, a marker of T-cell exhaustion. Interestingly, blocking CD39 with a nanobody in CD8^+^T-cells reverted its dysfunction, increasing cytotoxic cytokine production, activation, and cell proliferation.^63^

 To overcome the effect of ADO in the TME, the inhibition of enzymes involved in its synthesis has been assayed. One of the chemotherapeutic drugs used in OC is docetaxel (DTX), which inhibits the cell cycle by disrupting microtubules. DTX also induces CD73 overexpression, leading to ADO accumulation. Inhibition of CD73 using a monoclonal antibody was assayed together with DTX administration. Studies using syngeneic xenotransplantation of ID8 cells in mice showed that CD73 inhibition enhances the chemotherapeutic effects of DTX, as researchers observed a decrease in tumor growth and lung metastases.^64^ On the other hand, a clinical trial in patients with advanced OC evaluated the combined effect of two inhibitor antibodies: oleclumab, whose target is CD73, and durvalumab, which inhibits the immune checkpoint protein PDL1.^65^ The main objective of a phase II trial is to test the toxicity and efficacy of an anticancer drug or combination of drugs. The study by Mirza et al. demonstrated the tolerability and safety of administering a combination of antibodies targeting two important immune checkpoint proteins in patients. However, the anti-tumor activity was discrete. There may be several reasons for this; first, the trial’s inclusion criteria resulted in a heterogenous group with an advanced grade of the disease. Second, the sample size was relatively small. Third, the lack of racial diversity (92% of the patient population was white) limited the generalizability of the conclusions. A large, randomized trial comparing each antibody individually against the combination would be appropriate to clarify the findings. Table 1 summarizes the data described in this section.

**Table 1:** Summary of the purinergic elements (receptors or ectonucleotidases) and the subsequent effects in ovarian carcinoma.

**Purinergic element**	**Cell or Tissue**	**Effect**	**Ref.**
P2Y2	EFO-21, EFO-27	Showed two components in the calcium dynamic: a fast peak dependent on release from intracellular stores and a sustained component that involves influx.	^37^
	IOSE29, IOSE-29EC, OVCAR-3	Cell proliferation through the PKC and MAPK-ERK pathway.	^38^
	SKOV-3	EMT induction and cell migration by transactivation of EGFR.	^39^
		K^+^ channel KCa3.1 activates downstream of P2Y2 in the regulation of cell migration.	^40^
		Inhibition of SOCE decreases cell migration triggered by P2Y2.	^41^
P2X7	Macrophages	P2X7 can induce the NLRP3 inflammasome ensemble by extracellular ATP stimulation.	^47^
	HEK293	Upregulated expression of P2X7 in a group of biopsies of different organs.	^44^
	Carcinoma biopsies	P2X7 was upregulated and distributed mainly in the tumoral regions, induced Ca^2+^ influx, and its activation promoted cell proliferation and migration by phosphorylation of JNK, ERK, and AKT.	^45^
	Cancer in several tissues	Alternative splicing variants in the carboxyl terminus of the receptor modify its ability to induce cell death, and the effects depend on whether the receptor is fully or partially activated.	^32^
	OC biopsies	The components of the NLRP3 inflammasome and P2X7 receptor are overexpressed in omental adipocytes.	^48^
	SKOV-3, CAOV-3 and SW-626	Pharmacological inhibition or genetic knockdown reduced cell migration and vimentin levels and increased transepithelial resistance and E-cadherin expression.	^46^
A2B	SKOV-3	A2B signaling induces a decrease in cell migration and the presence of stress fibers and increases E-cadherin expression.	^53^
	OVCAR-3	A2B expression increases, and its activation induces cell survival and migration through a pathway involving IL-6 and Stat-3 activity.	^55^
CD73	Human biopsies from OC, ascitic body-derived cells, and OaW42 and SKOV-3	High expression and CD4^+^ and CD8^+^T-cell infiltration.	^56^
	Data from The Australian Ovarian Cancer Study (GSE9899)	A high expression level of CD73 is associated with a lower survival in patients.	^57^
	*In-vivo *model of OC	Inhibition of NAMPT and CD73 decreased the levels of ATP and NAD^+^ and reduced the tumor growth.	^58^
	SKOV-3	Showed limited ADO production by deficient conversion of ADP into AMP. Hydrolysis of extracellular ATP by apyrase reduces cell migration and expression of EMT cell markers.	^52^
	High-grade serous epithelial OC cells	Pharmacological inhibition of the ectonucleotidase decreases the formation of cell spheroids and tumorigenicity.	^60^
	Xenotransplantation of ID8 cells in mice	CD73 inhibition was shown to enhance the chemotherapeutic effects of DTX.	^64^
	Data from MetaGxOvarian dataset	High expression of both CD73 and CD39 in patients with high-grade serous carcinoma is related to a poor prognosis for patient survival.	^62^
	Patients with an advanced stage of OC	The combination of oleclumab and durvalumab showed limited clinical effectiveness as an antitumor effect.	^65^
CD39	Human biopsies from OC, ascitic body-derived cells, and OaW42 and SKOV-3	High expression and CD4^+^ and CD8^+^T-cell infiltration.	^56^
	Data from MetaGxOvarian dataset	High expression of CD39 and CD73 in patients with high-grade serous carcinoma is related to a poor prognosis for patient survival.	^62^
	CD8^+^T-cells	Blocking of CD39 with a nanobody, reversing its dysfunction, increasing production of cytotoxic cytokines, activation and cell proliferation	^63^

## A general perspective on purinergic element expression in public databases

**Figure 4:**
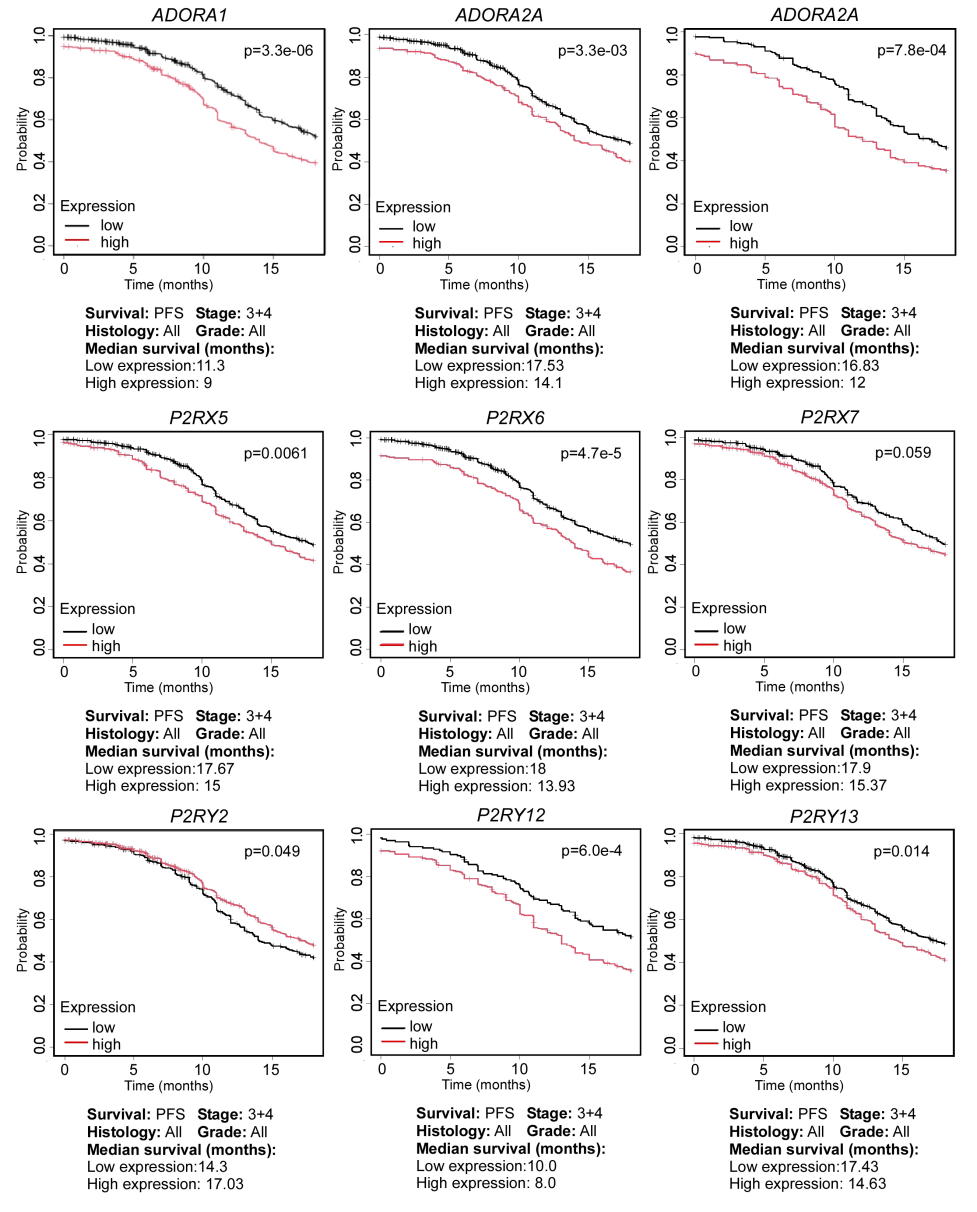
Effect of the expression of purinergic system elements and progression of ovarian carcinoma patients. Relationship between the expression levels of the purinergic elements (ADORA1, ADORA2A, ADORA3, P2RX5, P2RX6, P2RX7, P2RY2, P2RY12, and P2RY13) and progression-free survival (PFS), represented in means of median survival (in months). Data from the Kaplan Meier Plotter platform. Samples selected for this analysis were all types of histology, stages 3 and 4, all grades of disease. Missing purinergic elements did not meet a *P* < 0.05

 The experimental work described above highlights the feasibility of the purinergic system as a potential target in OC. This notion is also supported by data from publicly available databases. To denote the relevance of purinergic elements in OC, we used the *Kaplan-Meier Plotter for Ovarian Cancer (KMPOC)*^66^ to evaluate the expression levels of transcripts encoding for purinergic elements and their impact on the clinical outcome in terms of progression-free survival (PFS)—the duration from initial treatment until disease recurrence or progression. To enhance the relevance of our analysis to clinical settings, we evaluated PFS at 18 months, as 80% of relapses occur within this timeframe.^67^ For this analysis, we focused on patients diagnosed at stages 3 and 4, given that most OC patients are diagnosed at late stages,^68^ utilizing a sample size of 1081 patients. Our analysis focused on transcripts coding for purinergic receptors (P1 and P2) and CD73 and CD39 ectonucleotidases. The results, shown in Figure 4, indicate that purinergic elements play a significant role in the incidence of OC at 18 months. It is interesting to note that the lower the expression of most purinergic elements (specifically *ADORA1*, *ADORA2A*, *ADORA3*, *P2RX5*, *P2RX6*, *P2RX7*, *P2RY12*, and *P2RY13*), the slower the relapse of ovarian tumors. This finding contrasts with *P2RY2*, where lower expression correlates with quicker relapse (Figure 4). This suggests that purinergic elements promote tumor growth and recurrence, specifically at late stages.

 To further investigate the role of purinergic elements in OC, we explored the Gene Expression Omnibus (GEO) database^69^ and utilized the GEO2R tool^69^ to evaluate the expression of purinergic receptors across various patient data sets, and provide a translational approach (Table 2). GEO2R is an interactive web tool designed to compare two or more groups of transcriptomic data stored in GEO to identify differentially expressed genes. GEO2R uses various packages from the Bioconductor project.^70^ To conduct our analysis, we searched for datasets where OC was compared to healthy tissue. Next, we set the Benjamini & Hochberg (false discovery rate) option for adjusting p-values (significant adjusted *P *value < 0.05); we only selected transcripts that met this threshold. Data are presented as log2 Fold Change (base-2 logarithm of the ratio of expression levels in the two conditions).

**Table 2:** Differentially expressed genes of purinergic elements in patient-derived samples

	**Ovarian serous carcinoma (n=61) vs benign sample (n=11)**		**Ovarian tumor (n=8) vs healthy fallopian tissue (n=8)**		**Ascites (n=27) vs benign sample (n=11)**	
**Accession number**	**GSE143897**		**GSE137238**		**GSE143897**	
**Gene**	**Adj. ** ***P*** **-value**	**log2FC**	**Adj. ** ***P*** **-value**	**log2FC**	**Adj. ** ***P*** **-value**	**log2FC**
*ADORA1*	9.41E-34	4.967342	1.49E-02	1.971163	3.07E-38	5.925104
*ADORA2A*			3.45E-02	-1.06686		
*ADORA2B*	1.03E-02	1.032854			3.47E-06	1.878248
*ADORA3*	1.09E-16	3.084616			2.99E-09	3.63454
*P2RY1*	5.29E-08	-2.26591			8.75E-10	-2.67261
*P2RY2*	3.44E-18	3.503569			6.77E-19	3.546124
*P2RY6*	5.40E-32	4.073296	4.28E-03	1.734082	5.80E-28	4.699851
*P2RY12*			5.03E-02	-1.52071		
*P2RY13*	4.06E-03	1.130347	1.03E-03	-1.1916		
*P2RY14*			4.52E-05	-3.43825		
*P2RX1*			1.07E-08	-4.53303		
*P2RX3*	2.62E-04	3.314703				
*P2RX5*	2.19E-08	3.176583			2.65E-12	3.592917
*P2RX6*					2.41E-03	-1.11167
*P2RX7*	2.01E-04	-1.09121				

Data are presented as means of log2 Fold Change (log2FC). Ovarian carcinoma samples (n = 61) were compared to benign non-specific tissue (n = 11), accession number GSE143897. Ovarian tumor samples (n = 8) were compared to healthy fallopian tube tissue (n = 8), accession number GSE137238. Ascite-derived samples (n = 27) were compared to benign non-specific tissue (n = 11), accession number GSE143897. Blank spaces indicate genes that were not found in the transcriptomic analysis or whose adjusted p-value was higher than 0.05. Using the GEO database and GEO2R tool, we conducted a search for genes that exhibited significant differences in expression levels (adjusted *P*-value < 0.05) from samples derived from patients

 We used data from accession number GSE143897 to compare OC samples (n = 61) to benign non-specific tissue (n = 11). We observed up-regulation of *ADORA1*, *ADORA2B*, *ADORA3*, *P2RY2*, *P2RY6*, *P2RY13*, *P2RX3, *and *P2RX5 *(log2FC of 4.97, 1.03, 3.08, 3.5, 4.7, 1.13, 3.31 and 3.18, respectively), and down-regulation of *P2RY1* and *P2RX7 *(log2FC of -2.27 and -1.09, respectively) in tumor tissue compared to healthy tissue. In the same context, using data from accession number GSE137238, we assessed ovarian tumor samples (n = 8) from healthy fallopian tube tissue (n = 8), observing increased expression of *ADORA1 *and *P2RY6* (log2FC of 1.97 and 1.73, respectively) and down-regulation of *ADORA2A*, *P2RY12*, *P2RY13*, *P2RY14, *and *P2RX1* (log2FC of -1.07, -1.52, -1.19, -3.44, and -4.53, respectively) in the tumor samples in comparison to healthy tissue. We also evaluated ascite-derived samples (n = 27) compared to benign non-specific tissue (n = 11) using data from accession number GSE143897. Our findings indicated an up-regulation of *ADORA1*, *ADORA2B*, *ADORA3*, *P2RY2*, *P2RY6, *and *P2RX5 *(log2FC of 5.92, 1.88, 3.55, 4.70, and 3.59, respectively) and a down-regulation of *P2RY1 *and *P2RX6* (log2FC of -2.67 and -1.11, respectively) in the ascites in comparison to benign tissue.

Table 2 provides a summary of all generated and analyzed data, highlighting only significantly expressed results with an adjusted p-value below 0.05. The findings suggest that an overexpression of *ADORA1*, *ADORA2B*, *ADORA3*, *P2RY2*, *P2RY6, *and *P2RX5 *transcripts is required for tumor progression, at least at certain stages of ovarian carcinoma development.

 Each dataset represents a particular condition. Genes encoding purinergic elements are identified as transcriptional targets in the cellular changes that cause healthy cells to turn cancerous. However, it is essential to experimentally assess changes at the protein level. This type of informational data provides an excellent framework for proposing specific research programs centered on purinergic signaling. The variation in gene regulation across different systems underscores the complexity of the purinergic system and highlights its significance in the study of a multifaceted disease like OC.

## Conclusion

 Purinergic signaling has been identified as an important intercellular communication system in the TME. Extensive research supports its role in the evasion of host immune attacks (mainly mediated by the ADO/A2A receptor) and in various autocrine-paracrine actions that modulate cellular processes, such as the induction and maintenance of an invasive phenotype and cell proliferation (P2Y and P2X receptors). Our analysis of transcriptomic data from public databases suggests a correlation between purinergic element expression levels and patient prognosis. Thus, taken together, the reviewed data indicate that purinergic signaling proteins are potential therapeutic targets and disease markers.

## Competing Interests

 None to declare.

## Ethical Approval

 Not applicable.
